# Hôpital Beaujon

**Published:** 1829-04-01

**Authors:** 


### XXXVI.

### HOPITAL BEAUJON.

1. CONTUSION OF THE PERINEUM—FRACTURE AND SEPARATION OF THE PELVIS—DEATH.*

Prior to giving the details of this case, M. Blandin, the very intelligent reporter, furnishes a general, yet good anatomical description of the pelvic fasciæ. These fasciæ are a great deal talked of by our English HOSPITAL REPORTERS, but appear to be little understood, and, therefore, we think we should be doing some service by briefly running over M. Blandin's description.

The outlet of the pelvis is guarded by a triple fascia. The first is that immediately exterior to the peritoneum, continuous with the fascia iliaca, and fascia transversalis, investing the bladder and separating it from the peritoneum. With the middle pelvic fascia this forms a kind of sheath, in which we discover the two first portions of the urethra and the prostate, whilst the third or lowermost fascial aponeurosis adheres to the rami of the ischium and pubes, and contains in its embrace the bulb of the urethra and commencement of its spongy portion. The influence exercised by these pelvic fasciæ on affections of the region in which they are found must be evidently very considerable. For instance, if the bladder is burst, the urine which it contains is effused into the cellular membrane in contact with the peritoneum, and a fatal peritonitis is the obvious result. If, however, the urine escapes from the bulb of the urethra, it is prevented, of course, from passing backwards and is driven into the scrotum, perineum, penis, &c. as we commonly find in cases of extravasation of urine in consequence of strictures of the urethra. Suppose, again, that the pubes has been fractured, and the middle fascia of the pelvis destroyed, the fluids effused accumulate at first in the neighbourhood of the fracture, and are prevented by the *superior* fascia, which remains, from arriving at the peritoneum. After a little time, the middle aponeurosis being destroyed, the fluids pass downwards under the *inferior* to the scrotum, leaving the region of the anus intact. We now proceed to the narration of the case which illustrates and gave rise to the above remarks.

A child, about twelve years of age, was knocked down by a voiture, the wheel of which passed across the belly. This was on the first of September, 1828, and next day the patient was brought to the Hôpital Beaujon. There was great pain on pressure about the part, or on attempting to move the lower extremities, and the scrotum, perineum, hypogas-

* *Journal Hebdomadaire. No.* 8.

trium, and right haunch, were considerably swollen, and of violet colour. M.M. Marjolin and Blandin suspecting a fracture of the pubes, ordered a bleeding, and the application of rags dipped in lotion. In the evening of the 2d, the patient having made no water since the accident, the catheter was introduced, and the urethra found to be compressed and narrowed by the fluids effused around it. The bleeding was repeated, but the swelling in the perineum had augmented next day, and the pulse was quicker and hard. On the 4th, a deep fluctuation being felt in the perineum, an incision was cautiously made on the left side of the raphé, when there issued a gush of reddish-coloured fluid, followed by a liquid, looking like urine, but having no urinous smell. On pressing the abdomen this fluid was discharged with augmented force, and many who were present thought that the bladder had been wounded, although M. Blandin himself, for reasons that we need not stop to detail, entertained a different opinion. To demonstrate that the opening in perineo and interior of the bladder had no communication, M. Blandin, on the 5th, introduced a sound into the latter and a probe into the former, without being able to make them communicate or touch one another. On the 6th, fluctuation being found in the right groin, an incision was made and purulent matter let out. Little relief was obtained; another incision was required in the loins; diarrhœa supervened; the fluid which escaped from the wound in the perineum was first tinged with pus, and then presented a decidedly urinous odour; and, at length, on the morning of the 9th, the patient breathed his last.

*Dissection.* The fascia iliaca and cellular membrane immediately external to the peritoneum in the pelvis was injected with dark-coloured blood. The bladder was sound, the superior pelvic fascia uninjured, but raised on the right side by a vast collection of dark putrid pus, which extended from the right sacro-iliac synchondrosis, by the side of the levator ani, to the part of the perineum where the incision had been made. The matter pervaded the sacro-iliac joint, the bones being apart and bathed in pus. The ilium was fractured in two situations, the symphysis pubis destroyed, and matter which had formed around the fractured bones communicated with the larger abscess above-described. The middle perineal fascia on the right side was broken down, and permitted the passage of pus to the inferior. The abscess had not passed at all by the side of the rectum, but the membranous part of the urethra and bulb were exposed in its centre, bare, and deprived of their cellular tissue. The membranous portion was softened, and presented a small, circumscribed, and recent perforation, through which had issued the urinous fluid noticed towards the termination of the case. The inferior perineal fascia was sound, and had bounded the deep-seated abscess until the incision had been made in it by the knife. The viscera, &c. were sound.

The above is an exceedingly interesting case, at least to our surgical brethren, and exemplifies the influence exerted on collections of fluid in the outlet of the pelvis by the perineal fasciæ. A question arises, which is not set at rest by the report—from whence did this great effusion in the first instance proceed? We have heard of a case which happened in one of our metropolitan hospitals, where a surgeon made incisions for what he considered extravasated urine, but which afterwards proved to be rupture of the femoral vein in consequence of fracture of the pelvis. A great deal of difficulty frequently exists in distinguishing the actual nature of these cases, for not long ago we witnessed an instance where rupture of the membranous part of the urethra, and extravasation of urine into the cellular membrane, behind and above the pubes, was never suspected by some very eminent surgeons during life.

II. Canceroid Ulcer of the Upper Lip, successfully treated by Antiphlogistics and Caustics.

Jean Sagite, ætatis 65, of sanguineous temperament, entered the Hôpital Beaujon on the 6th of May, 1828, on account of an ulcer in the upper lip, immediately beneath the left ala nasi. The sore was an inch in circumference, and half an inch in breadth—its edges were ragged—its surface of a dirty grey with reddish and easily bleeding granulations in its centre. The parts affected appeared to be the skin, the muscle, and a small portion of the mucous membrane, and the lip to the extent of two inches around was triple its natural size. The patient had been affected with a fissure in the lip for two months before his admission, but this had not increased or produced inconvenience till within the few days immediately preceding it.

Ten leeches were applied on the 8th, and afterwards a poultice. The leeches were repeated on the 10th, the 15th, and the 16th, whilst the diet consisted entirely in vegetable broths and ptisans. The lip soon regained its natural size, but the ulceration, though superficial, appeared to spread, and the surface was touched with the nitrate of mercury on the 26th. The cauterization was repeated on the 30th, and again on the 3d of June, after which it was no more required. The lip had lost its induration, the ulcer healed, and the patient was discharged cured on the 13th.

M. Petit, the "interne" who reports the case, observes that by many the above would have been called cancer and been removed by the knife. Many such cases have been cured at the Hôpital Beaujon by ordinary treatment, especially antiphlogistics, followed by cauterization. The former removes the swelling and inflammation set up around, and the latter converts an irritable sore into a simple suppurating wound, readily disposed to cicatrization. There are very many ulcers, however on the lip that obstinately resist all local applications, but yield to an alterative course of mercury, especially given in the form of the oxy-muriate. The following case which occurred at St. George's Hospital, is valuable in a therapeutical view.

Mary Ann Voy, a healthy looking girl, ætatis 18, was admitted Oct. 22d, 1828, under the care of Mr. Brodie, with an affection of the under lip, of which she furnished the following account. She had noticed since childhood a small, dark-red, and slightly elevated spot, most probably a nævus, on the centre of the under lip. Two years ago this burst out bleeding, which recurred at intervals for a fortnight, or more, when an ulcer was the consequence. This ulcer often bled, continued to extend, and resisted every mode of treatment had recourse to. Amongst other remedies, mercury was tried, but it failed to affect the mouth, and otherwise did no good. A year before her admission into St. George's, she entered Guy's Hospital, where various medicines, and various local applications were again ineffectually tried. During her stay at Guy's, a tumour appeared at the junction of the lip and the gum, which increased to the size of a nut, and was then removed by Mr. Cooper, together with the ulcer on the lip, by an operation. The whole wound healed in the course of a month, and since that time the cicatrix on the lip had continued sound, whilst that next the gum, had, on one or two occasions given way and again healed up. Two months before her admission, it broke out afresh, and had not again cicatrized.

On admission there was noticed a shallow, narrow, dirty-looking ulcer an inch in length, at the commissure between the gum and the lower lip. The edges were neither everted nor hard, they were scarcely, if at all, elevated, and indeed the ulcer had no well marked character of scirrhus. She complained of a little, but not much, pain—the lip was everted —the chin slightly swollen—catamenia regular—rest at nights disturbed—appearance, as we mentioned before, not unhealthy.

The nitrate of silver was applied to the ulcer, and Nov. 1st, she was ordered a drachm of the liquor hydrargyri oxymuriatis, a drachm of the compound tincture of cinnamon, and ten drachms of distilled water three times a day. On the 28th, the oxymuriate was beginning to affect the system, for the patient complained of loss of appetite, looked pale, and felt sick. The ulcer, however, had greatly improved, being perfectly clean, and almost healed. Pressure was now had recourse to, and after the expiration of a week or ten days, it seemed to produce a good effect, as the ulcer very rapidly healed, and the patient was dismissed the hospital cured.

It is worthy of remark, that this young woman had several times taken mercury and sarsaparilla without any benefit. On no previous occasion, however, had the mouth or the system been affected, whereas the oxymuriate exhibited in the present instance had an evident constitutional effect. For our own parts, we believe that the best mode of exhibiting the mercury in these foul, or pseudo-malignant ulcers, is by means of the oxymuriate, either in tincture of bark, or, as here, with aromatics.

---

### XXXVII.

### GLOUCESTER INFIRMARY.*

### I. DISLOCATION OF THE FEMUR INTO THE ISCHIATIC NOTCH.

### HOPITAL BEAUJON.

HIGH OPERATION FOR THE STONE—CALCULUS NOT EXTRACTED—RECTO-VESICAL OPERATION PERFORMED—DEATH OF THE PATIENT.*

The vulgar have a saying, that "it never rains but it pours," and the vulgar are certainly right. If a difficult or curious case of hernia is recorded, the medical journals, weekly, monthly, and quarterly, are filled with herniæ, till a *rupture* is almost brought about between the writers of the said journals and the readers. Since Mr. B. Cooper's case of lithotomy, the press has teemed with other and similar cases, and ten to one, if a medical man opens a periodical publication, but he is *bored* with details of the *lithontripteur*, or, like the Apostle, narrowly escapes being *stoned* to death! The case, however, of which we are about to furnish a brief account, is by no means an every-day one, and appears to have excited some éclât amongst our Parisian confrères.

A lad of 15, apparently worn to the bone with suffering, was admitted into the Hôpital Beaujon, on the 1st of December last, with all the symptoms of stone in the bladder, symptoms under which he had laboured for about five years. On sounding him, which produced excessive pain, M. Blandin pronounced the existence of a very large calculus, resting on the neck of the bladder, and pushing it back from the side of the perineum. The finger introduced into the rectum confirmed this opinion, and discovered the calculus pressing the fundus of the bladder considerably downwards. On the next day M. Marjolin repeated the examination, and arrived at a similar conclusion, adding, that the stone extended transversely from one tuberosity of the ischium to the other. Under these circumstances, the surgeons were convinced that nothing but the high operation could succeed, and, after some preparatory treatment, this was performed, on Sunday, December 7th, before a large concourse of students, surgeons, and physicians.

The steps of the operation we need not

* Journal Hebdomadaire, Nos. 11-12.

minutely describe, suffice it to say, that after the bladder had been as much injected as the size of the stone, and its contraction, would allow, it was cautiously, and not without considerable difficulty, cut into from above the pubes, the suspensory hook (le crochet suspenseur) placed in the upper part of the wound in the viscus, and the forceps introduced upon the finger. The stone was touched, but it could not be moved; and, despite of a variety of very ingenious and patient trials, there it remained, at the neck of the bladder, as firm as a rock. Another moderately-sized calculus, situated above, was extracted. At length, when all attempts to dislodge the stone had proved ineffectual, and the surgeons were afraid of exhausting the patient by further attempts, which, in all probability, would not have succeeded, M. Blandin, with the advice and concurrence of his colleagues, determined to extract it, if he could, from below. Having introduced into the rectum the index finger of the left hand, till it felt the projecting bladder and stone, a straight and sharp-pointed bistoury was carried upon it, till it reached the same spot, when the point was turned up, and the neck of the bladder, the prostate, the anterior wall of the rectum and anus, and the neighbouring part of the perineum, divided in the median line, by drawing the bistoury out. After some useless trials to dislodge the stone, this was finally done, by an assistant thrusting it downwards with his finger from the wound above the pubes, whilst tractions were made at the same instant by the forceps from below. The operation was very long and laborious, but the patient lost little blood. The calculus was two inches and a line in length, and sixteen lines in its shortest diameter. The wound in the hypogastrium was closed by bandages, &c. but that below left open for the urine to escape. He was bled in the evening, and again in the morning, although no untoward symptoms appeared till next evening, when the pulse was 110, and the patient complained of pain in the hypogastrium. The symptoms of peritoneal inflammation made progress—the urine did not flow with freedom, and a seton was passed between the perineal and hypogastric wounds, from the latter of which the bandages were removed—but, on the third day after the operation, the patient died.

On the dissection, which was made in the presence of most of those who had witnessed the operation, the whole internal surface of the peritoneum was found to be more or less acutely inflamed, although it had not been wounded in either operation. The hypogastric incision in the bladder extended from its apex to the prostate—the perineal comprehended the skin, the sphincter ani in front, the anterior wall of the rectum, for an inch and a half above the sphincter, the membranous portion of the urethra, the prostate gland, and the part of the urethra within it. The neck of the bladder was uncut. The walls of this viscus were greatly thickened, the mucous membrane bore marks of chronic inflammation, and the organ adhered at its apex, by indurated cellular membrane, to the walls of the abdomen. The stone had been lodged in an enormous dilatation of the prostatic part of the urethra, which dilatation had been divided by the lower incision. The cellular membrane throughout the pelvis was infiltrated with pus and urine, which was also found behind the peritoneum, along the psoæ, even to the loins. The ureters, with the pelvis and infundibula of the kidneys, were extremely dilated, and the renal substance itself half wasted.

This was one of those cases which, no doubt, would have terminated badly with any operator, or under any operation; at the same time, it has not tended to lessen the dislike we entertain towards the "high operation," a dislike not founded in theory or prejudice, but bottomed on what we have read and what we have *seen*. We witnessed this operation twice at St. George's Hospital, and certainly our minds received at the time a very strong bias against it. Tedious, painful, difficult and dangerous, it has scarcely an advantage which the lateral operation does not equally possess, and many disadvantages peculiar to itself. Setting aside the danger of wounding the peritoneum, the surgeon has every thing to dread from urinous effusion into the cellular membrane of the pelvis, an effusion which proved fatal to the present patient, and *has* proved fatal to many. Our lively neighbours, the French, who are easily led away by a new proposal, or, indeed, by any thing whatever, new or old, which carries with it éclât, and varies the monotony of established modes of practice, seem to have adopted the high operation with a degree of vivacity and perseverance which English surgeons have not displayed. It may succeed in some few cases, it may even, under peculiar circumstances, be preferable to the lateral operation; but we do contend that occasions for its employment are very rare, and repeat that it is not only more fatal, but also a great deal more tedious and *difficult* of performance than the latter. We suspect that the lodgment of a stone in the prostatic portion of the urethra is not extremely uncommon, at least we saw, a year or two ago, a well-marked instance of a calculus so placed. Had the high operation been performed in that case, we do not believe that the stone could possibly have been extracted.

---

### LVII

### ST. THOMAS'S HOSPITAL.

[MR. BURY, REPORTER.]

